# Casein Kinase 2 Interacting Protein-1 Suppresses Glioma Cell Proliferation via Regulating the AKT/GSK3*β*/*β*-Catenin Pathway

**DOI:** 10.1155/2019/5653212

**Published:** 2019-07-02

**Authors:** Yan-Guo Xi, Deng-Peng Ren, Jing-Yun Jin, Lei Zhu, Tai-Long Yi, Xue-Fei Shao, Sheng-Kai Sun, Wen-Bin Zhang, Shi-Xiang Cheng

**Affiliations:** ^1^Institute of TBI and Neuroscience, Characteristic Medical Center of Chinese People's Armed Police Force (PAP), Tianjin Key Laboratory of Neurotrauma Repair, No. 220 ChengLin Road, HeDong District, Tianjin 300162, China; ^2^Department of Neurosurgery, Cangzhou Central Hospital, No. 16 XinHua West Road, YunHe District, Hebei 061001, China; ^3^Department of Neurosurgery, YunCheng Central Hospital, No. 3690 HeDong East Road, YanHu District, Shanxi 044000, China; ^4^Department of Neurosurgery, Yi-Ji Shan Hospital of Wannan Medical College, No. 2 ZhenShan West Road, Wuhu, Anhui 241000, China

## Abstract

**Objective:**

Casein kinase 2 interacting protein-1 (CKIP-1) has exhibited multiple functions in regulating cell proliferation, apoptosis, differentiation, and cytoskeleton. CKIP-1 also plays an important role as a critical regulator in tumorigenesis. The aim of this study is to further examine the function of CKIP-1 in glioma cells.

**Methods:**

The expression level of CKIP-1 protein was determined in gliomas tissues and cell lines by immunohistochemistry stain and western blotting while the association of CKIP-1 expression with prognosis was analyzed by Kaplan-Meier method and compared by log-rank test. CKIP-1 was overexpressed or silenced in gliomas cell lines. CCK-8, colony formation assay, and BrdU incorporation assay were used to determine cell proliferation and DNA synthesis. Cell cycle and apoptosis rate were determined with fluorescence-activated cell sorting (FACS) method. Then, expression of key members in AKT/GSK3*β*/*β*-catenin pathway was detected by western blot analysis.

**Results:**

In the present study, we reported new evidence that CKIP-1 was reversely associated with the proliferation of glioma cells and survival in glioma patients. Additionally, the overexpressed CKIP-1 significantly inhibited glioma cell proliferation. Further experiments revealed that CKIP-1 functioned through its antiproliferative and proapoptotic activity in glioma cells. Importantly, mechanistic investigations suggested that CKIP-1 sharply suppressed the activity of AKT by inhibiting the phosphorylation, markedly downregulated the phosphorylated GSK3*β* at Ser9, and promoted *β*-catenin degradation.

**Conclusions:**

Overall, our results provided new insights into the clinical significance and molecular mechanism of CKIP-1 in glioma, which indicated CKIP1 might function as a therapeutic target for clinical treatment of glioma.

## 1. Introduction

In the central nervous system, gliomas are the most common type of aggressive primary solid tumor, accounting for ~80% of primary malignant brain tumors. The incidence of gliomas has increased in recent years and is still with significant mortality [1]. Malignant glioma is characterized by invasion into surrounding brain tissues, rapid proliferation, and aberrant angiogenesis [2]. These biological characteristics lead to unclear boundaries between tumors and surrounding brain tissues and the glioma cells usually spread deep into the brain which pose a great challenge to surgeons. Although multiple therapies include maximum safe resection, followed by chemotherapy, radiation, and immunotherapy, the outcome of patients diagnosed with high-grade glioma remains unsatisfied, with median survival of less than 15 months [3, 4]. Therefore, it is vital to investigate the specific molecular mechanism that regulate the progression of glioma and identify the novel molecular therapeutic targets to aid in development of glioma therapies.

The pleckstrin homology (PH) domain-containing protein casein kinase 2 interacting protein-1 (PLEKHO1, also known as CKIP-1) was originally identified as an interactive partner of the casein kinase 2 (CK2) *α* subunit [5]. The* CKIP-1* gene encodes a 409 amino acid protein which contains a PH domain at the N-terminus, as well as five proline-rich motifs at the middle region and leucine zipper (LZ) motif at the C-terminus [6]. The PH domain is critical for the CKIP-1 protein's subcellular localization and regulates protein activity, protein-lipid, and protein-protein interactions during multiple signal pathways [7, 8]. Previous studies reported that CKIP-1 could modulate cell growth, apoptosis, cytoskeleton, differentiation, and bone formation by interacting with multiple proteins, such as AP-1/c-Jun, CK2*α*, Akt, and Smurf1 [9–12]. In addition, a growing body of literature demonstrates that CKIP-1 was highly expressed in normal tissues, but extremely lower in some cancer tissues and cancer cell lines, including lung cancer, breast cancer, osteosarcoma, ovary cancer, and colorectal cancer, suggesting the potential tumor suppression ability of CKIP-1 [13, 14]. However, few studies examining the function of CKIP-1 in glioma cells have been performed.

In our previous experiments we found that CKIP-1 and glycogen synthase kinase-3*β* (GSK-3*β*) were all low expressed in human glioma U251 cells and human glioma tissues. GSK-3*β*, a widely expressed serine/threonine kinase, is an important cytosolic negative regulator of the Wnt pathway. GSK-3*β* inhibits *β*-catenin cytosolic stabilization and the process of nuclear migration. Although it was pointed out previously that GSK-3*β* dysregulation was associated with glioma genesis and progression, the function of CKIP-1 and the relationship between CKIP-1 and GSK-3*β* in glioma remained to be elucidated. In this report, we investigated the effects of CKIP-1 on glioma cells and explored the molecular mechanisms behind the growth-suppressive activity of CKIP-1 associated with GSK-3*β* in glioma cells.

## 2. Materials and Methods

### 2.1. Cell Culture and Reagents

Human glioblastoma cells (U251, U87, A172, and U373) were obtained from the Cell Bank of Chinese Academy of Science (Shanghai, China). All cell lines were maintained in Dulbecco's modified Eagle's medium (DMEM, Hyclone, USA) supplemented with 10% (*v/v*) fetal bovine serum (FBS, Gibco) and 1% penicillin/streptomycin at 37°C with 5% CO_2_.

### 2.2. Tissue Specimens

This study was approved by the ethics committee of Characteristic Medical Center of Chinese People's Armed Police Force. With Institutional Review Board approval, 8 normal human brain tissues from accident victims and 87 freshly resected human glioma specimens (pathologically confirmed: WHO grades 1-4) were collected from the Characteristic Medical Center of PAP and Department of Neurosurgery of Cangzhou Central Hospital between January 2010 and December 2017 with written informed consent from the patients with a newly diagnosed glioma who had not received therapy before sample collection. Detailed patient information was shown in Table 1.

### 2.3. Overall Survival (OS) Analysis

A cohort of 87 glioma patients was divided into* CKIP-*1_low_ and* CKIP-*1_high_ based on* CKIP-1 *mRNA levels using mean* CKIP-1* mRNA levels of all the patients as the denominator. OS analysis was performed using a standard Kaplan-Meier curve.

### 2.4. Reverse Transcriptase-Polymerase Chain Reaction (RT-PCR)

Total RNA was extracted with TRIZOL reagent according to the manufacturer's instructions. 2 *μ*g of total RNA was used to perform reverse transcribed by using M-MLV-RTase and oligo dT following the manufacturer recommendations (Promega). The following primers were used: 5′-ATC ACC CGA GCC AAG AAC C-3′ (forward primer) and 5′-GGA AGC CAC AGC CAT TAG G-3′ (reverse primer) (141 bp) for human CKIP-1, and 5′-TGA CTT CAA CAG CGA CAC CCA-3′ (forward primer) and 5′-CAC CCT GTT GCT GTA GCC AAA-3′ (reverse primer) (121 bp) for human glyceraldehyde-3-phosphate dehydrogenase (GAPDH).

### 2.5. Plasmid and Stable Transfection of Glioma Cells

The ORF sequence human* CKIP-1* (GenBank No. NM_016274) cDNA was isolated from U-87MG cells by RT-PCR and confirmed by DNA sequencing and wan then cloned into* Age* I and* EcoR* I sites of Flag-tagged mammalian expression vector pcDNA3.1 (Invitrogen, USA). The siRNA sequence targeted to the* CKIP-1* (5′- GAG CTG AGA GAC CTG TAC AGA-3′) was cloned into the lentiviral siRNA expressing vector pGCSIL-eGFP (GeneChem, China) and confirmed by DNA sequencing. Cells were cultured in 6-well plates and then transfected with pcDNA3.1-CKIP-1 or pcDNA3.1-empty vector plasmids, or pGCSIL-CKIP-1 or pGCSIL-Ctrl at a density to ensure ~80% confluence. Transfection was performed using Lipofectamine 3000 reagent (Invitrogen) according to manufacturer's instructions with a DNA to Lipofectamine ratio of 1:3 w/v.

### 2.6. Cell Proliferation Assay

Cell proliferation was evaluated by using Cell Counting Kit-8 (CCK-8) (Sigma, USA), according to the manufacturer instructions. In brief, cells (3 × 10^3^/well) were seeded onto the 96-well plate. For detecting the relative cell proliferation rate, CCK-8 reagent was added to each well and allowed to incubate for 3 h at 37°C. Then, the absorbance value (*A*) at 490 nm was measured by the microplate reader. Additionally, viable cell number was counted with Cellomics Array-Scan™ VTI HCS Reader (Thermo Fisher, USA) according to the supplier's recommendations.

### 2.7. 5-Bromo-2′-Deoxyuridine (BrdU) Assay

Cells (1 × 10^3^/well) were seeded into a 96-well plate and incubated for 24 or 96 h, and 10 *μ*l of BrdU (Thermo Fisher, USA) reagent was then added in each well from 2 to 24 h followed by adding 100 *μ*l fixing solution for 30 min. Cells were washed with washing buffer and labeled with anti-BrdU antibody (Thermo Fisher, USA) for 12 h according to the manufacturer's instruction. Pipette 100 *μ*l/well 1 × Peroxidase Coat Anti-Mouse IgG (Abcam, UK) was conjugated and incubated for 30 min at room temperature. After washing the plate with wash buffer, 100 *μ*l TMB peroxidase substrate was added in each well and incubated for 30 min at room temperature in the dark. The absorbance value was measured by the microplate reader at 450 nm.

### 2.8. Colony Formation Assay

Cells in a logarithmic growth phase (1 × 10^3^/well) were plated into 6-well plates. After being incubated in DMEM with 10% FBS for two weeks, cells were washed with phosphate-buffered saline (PBS) and fixed with methanol for 30 min and colonies were stained with 0.1% crystal violet and photographed. The number of clones (50 cells or more were considered a colony) was counted.

### 2.9. Cell Cycle and Apoptosis Assays

For cell cycle assay, cells in a logarithmic growth phase were collected and fixed with 70% ice-cold ethanol overnight at 4°C. After centrifugation, cells were stained with 100 mg/l RNase and 5 g/l propidium iodide (PI) in the dark for 30 min at room temperature. Detection of cell cycle distribution in G_0_/G_1_, S, and G_2_/M phases was then performed on a BD FACSCalibur. FCS Express Version 3.0 software was used to analyze the data.

For cell apoptosis assay, Annexin V-fluorescein isothiocyanate (FITC)/PI staining was performed using the Annexin V-FITC/PI Detection Kit (BD Biosciences, USA) according to the manufacturer's instructions. Briefly, the cell suspension was incubated with 5 *μ*l Annexin V-FITC and 10 *μ*l PI for 30 min at room temperature in the dark; then the apoptotic cells were counted by flow cytometer. All experiments were performed in triplicate.

### 2.10. Immunohistochemistry (IHC)

Tissue sections were rehydrated in an ascending series of ethanol solution and then inhibited the endogenous peroxidase activity in 0.3% hydrogen peroxide. The sections were heated at 105°C in 10 mM sodium citrate (pH 6.0) for retrieving the antigen. Afterwards, nonspecific protein binding was blocked by 5% goat serum and then incubated with monoclonal anti-human CKIP-1 primary antibody at 4°C overnight and staining was visualized using the specific HRP/DAB Detection kit (eBioscience, USA) according to the manufacturer's instructions. Finally, the sections were rinsed in PBS and counterstained with hematoxylin. Staining results were evaluated by two pathologists in a blinded way. Five high-power fields were selected randomly and 300 cells in each field were counted to determine the labeling index (LI), which represents the estimated percentage of positive cells relative to the total cells. The proportion of positive cells was estimated and given a score on a scale from 0 to 3 (0 = 0% to 4%, 1 = 5% to 30%, 2 = 31% to 70%, and 3 = 71% to 100%). Staining intensity was estimated and given a score from 0 to 3 (0 = no staining, 1 = weak staining, 2 = intermediate staining, and 3 = strong staining). The scores of percentage and intensity were added as the final scores of 0-1 indicated negative expression (-), 2-3 indicated weak expression (+), 4-5 indicated moderate expression (++), and 6 indicated strong expression (+++). For statistical analysis, 0-3 were counted as low expression, and 4-6 were counted as overexpression. Normal human and glioma patients' brain tissues slides were used for exploring the expression of CKIP-1 and AKT/GSK3*β*/*β*-catenin.

### 2.11. Western Blotting

Cells were harvested and lysed in RIPA lysis buffer (Thermo Fisher Scientific, USA) for extracting total proteins. The protein concentrations were measured by a BCA protein assay kit (Transgen Biotech, China), separated by 12% sodium dodecyl sulfate polyacrylamide gel electrophoresis (SDS-PAGE) and transferred to nitrocellulose (NC) membranes. The membranes were then blocked by 10% nonfat milk and incubated with the corresponding antibody (CKIP-1, Akt, p-Akt, Gsk3*β*, *β*-catenin, p-*β*-catenin, Smurf1, and GAPDH; Abcam, Cambridge, MA, USA) diluted according to the instructions for 12 h at 4°C. After washing 3 times with PBS/0.1% Tween-20, membranes were incubated at room temperature for 2 h with horseradish peroxidase-conjugated secondary antibody (anti-mouse IgG and anti-rabbit IgG at 1:10000 dilution; Abcam). Protein expression levels were detected with ECL detection solution (Millipore, USA) and visualized with chemiluminescence detection system. The bands signal were analyzed using a gel imaging system (Bio-Rad).

### 2.12. Statistical Analysis

All statistical analysis was carried out with the software package SPSS 20.0 (SPSS, Inc., Chicago, IL). Data were presented as the mean ± standard error of the mean. Statistical significance was determined using a Student's* t*-test or one-way analysis of variance (ANOVA) followed by LSD test. Survival curves were plotted using the Kaplan-Meier method and compared with the log-rank test.* p* < 0.05 was considered to be statistically significant.

## 3. Results

### 3.1. Low Expression of CKIP-1 in Glioma Is Associated with a Poor Prognosis in Glioma Patients

**Figure 1:**
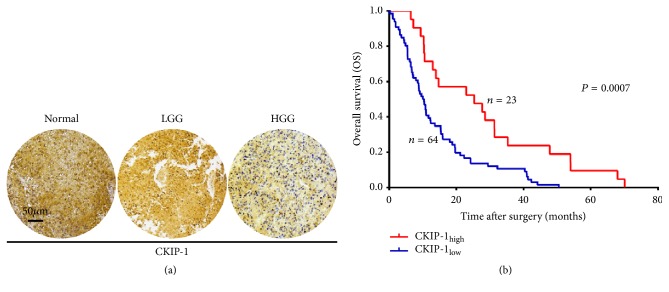
*CKIP-1 expression level inversely correlated with the survival of glioma patients*. (a) Representative photographs for CKIP-1 immunostaining in normal brain, LGG (WHO grades I-II), and HGG (WHO grades III-IV) tissues (scale bar = 40 *μ*m). (b) Kaplan-Meier analysis for patients with low CKIP-1 (CKIP-1_low_, blue line) versus high CKIP-1 expression (CKIP-1_high_, red line) in IHC analyses. LGG, low-grade glioma; HGG, high-grade glioma.

Given CKIP-1 being confirmed as a tumor suppressor in other types of tumors, we sought to determine the clinical relevance of the relation between CKIP-1 and gliomas. First, we performed immunohistochemical staining of tissue samples (87 glioma and 8 normal brain tissues) and examined the expression of CKIP-1 protein expression. We found CKIP-1 staining clearly was localized to the cytoplasm and nuclei in nontumor brain tissue (Figure 1(a)) and generally higher than glioma tissue. Immunoreactivity for CKIP-1 was noted in all 8 (100%) normal brain tissues. However, lower CKIP-1 expression levels of high-grade gliomas (HGG: WHO grades III-IV) are compared with low-grade gliomas (LGG: grades I-II, Figure 1(a)). Of a total of 87 glioma tissue samples, low expression of CKIP-1 accounted for 21.1% (4 of 19) in the LGG (grades I 3 and II 16) and 88.2% (60 of 68) in the HGG (grades III 27 and IV 41), respectively, and significantly correlated with WHO grade (*p* = 0.001, Table 1). Next, we divided glioma patient samples into two groups: CKIP-1_low_ and CKIP-1_high_ based on protein levels. For the analysis of OS (Figure 1(b)), the CKIP-1_low_ group had significantly poorer OS (14.0 ± 12.2 months) than the CKIP-1_high_ group (28.2 ± 20.0 months;* p* = 0.0007). These results demonstrated that lower CKIP-1 expression was sharply associated with poor survival in patients with gliomas, suggesting that CKIP-1 was probably a tumor suppressor in gliomas.

### 3.2. Altered CKIP-1 Expression Affected Glioma Cell Proliferation

**Figure 2:**
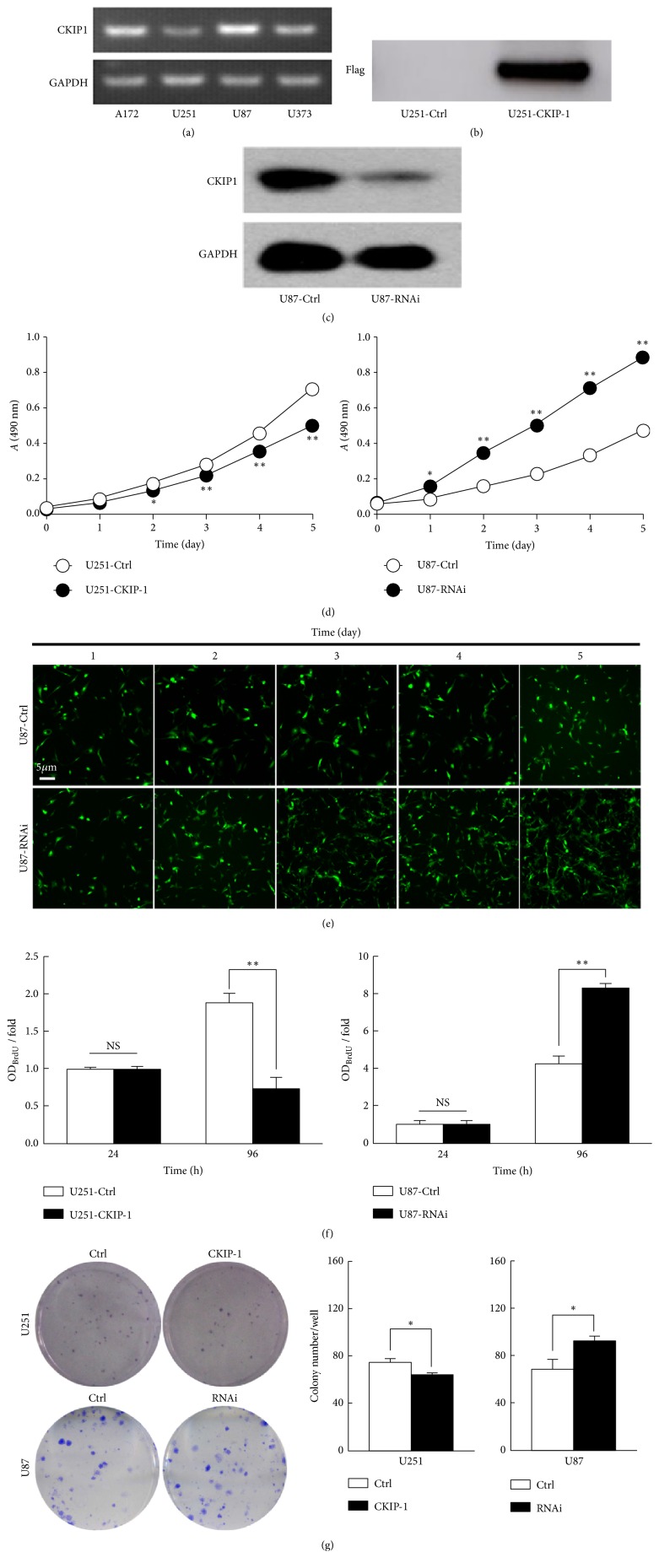
*Altered CKIP-1 expression affected glioma cell proliferation*. (a) Expression of CKIP-1 mRNA in four glioma cell lines. GAPDH was the loading control. (b) Overexpression of CKIP-1 in U251 cells by flag-tagged vector pcDNA3.1 for CKIP-1 (U251-CKIP-1) or empty vehicle (U251-Ctrl). (c) Cell proliferation was determined by Cell Counting Kit-8 (CCK-8) assay every day for 5 days. (d) Cell proliferation was assessed by 5′-bromodeoxyuridine (BrdU) incorporation 24 h and 96 h after transfection of glioma cells with vehicle (Ctrl), CKIP-1 (e). Cell growth of U251-Ctrl cells was assayed every day for 5 days. (f) Colony formation assay and quantification of U251 cells with CKIP-1 overexpression. *∗ p* < 0.05, *∗∗ p* < 0.01,* vs*. control.

To determine the CKIP-1 expression levels in glioma cell lines, we first investigated the CKIP-1 expression in U251, U87, A172, and U373 cell lines by RT-PCR. As shown in Figure 2(a), significantly lower expression of CKIP-1 was evident in U251 and U373 cells than in U87 and A172 cells. Then, CKIP-1 expression vector pcDNA3.1-Flag-CKIP-1 was transfected into the U251 cells which had low levels of CKIP-1 expression, established ectopic expression of CKIP-1 clones (U215-CKIP-1) as well as their vector control (U251-Ctrl), and was confirmed by the Flag antibody (Figure 2(b)). On the other hand, we transfected CKIP-1 siRNA into U87 cells, which typically express high levels of CKIP-1 and selected stable depleted CKIP-1 expression clones (U87-RNAi) as well as vector control (U87-Ctrl). As expected, the CKIP-1 expression was sharply decreased in U87-RNAi than that of control (Figure 2(c)).

We then asked whether altered CKIP-1 expression showed an effect on proliferation of glioma cells. Cell proliferation in stable clones overexpressing CKIP-1 or depleted CKIP-1 was evaluated by CCK-8 or fluorescence assay. During 5-day observation, we found that the proliferation rate of U251-CKIP-1 was significantly lower compared to U251-Ctrl (Figure 2(d), Left). Conversely, the proliferation rate of U87-RNAi was apparently higher than U87-Ctrl (Figure 2(d), Right). Consistent with this trend, the number of U87-RNAi cells increased more than ~10-fold, while the U87-Ctrl cells only increased ~4-fold (Figure 2(e)).

Next, we examined the proliferation of different glioma cells by performing BrdU incorporation assays (Figure 2(f)). We found that CKIP-1 induced a significant decrease of BrdU incorporation in U251 cells incubated for 96 h (U251-Ctrl* vs.* U251-CKIP-1 cells, mean fold-decrease = 1.9-* vs.* 0.7-fold;* p *< 0.01). In contrast, genetic knockdown of CKIP-1 sharply increased the BrdU incorporation in U87 cells (U87-Ctrl* vs. *U87-RNAi cells, mean fold-increase = 4.3-* vs.* 8.3-fold;* p* < 0.01).

Finally, colony formation assays were also used to investigate the role of CKIP-1 in glioma cell proliferation.  Figure 2(g) showed that the ability of U251 and U87 cells to form colonies was significantly altered when cells overexpressed or knocked down CKIP-1. Colony formation of U251 cells overexpressing CKIP-1 was reduced in contrast to increased colony number in U87 cells with CKIP-1 depletion. Collectively, these data demonstrated that CKIP-1 overexpression inhibited glioma cell proliferation, while CKIP-1 depletion showed increased tumor cell counts, suggesting that CKIP-1 may function as an antiproliferative factor.

### 3.3. Antiproliferative and Proapoptotic Activity of CKIP-1 in Glioma Cells

**Figure 3:**
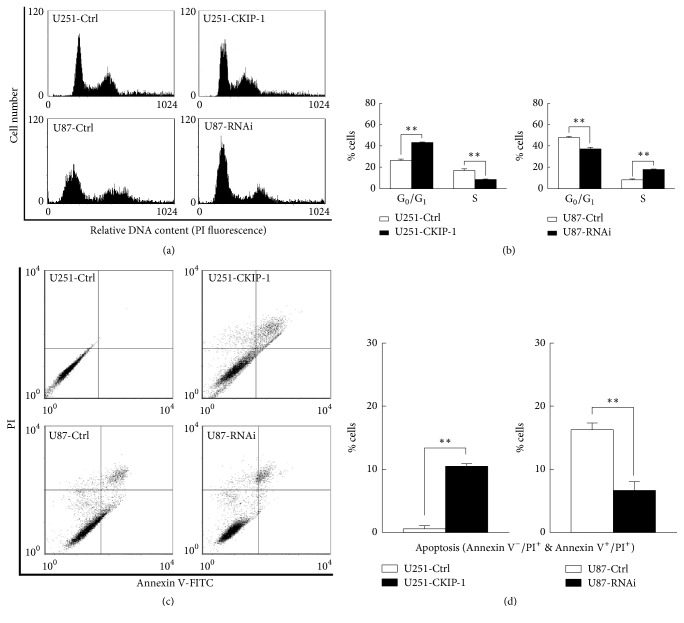
*Antiproliferative and proapoptotic activity of CKIP-1 in glioma cells*. (a) Cell cycle distributions were detected followed by propidium iodide (PI) staining in U251 cells transfected with CKIP-1 for 72 h by flow cytometry. (b) Cell cycle assays of percentage of cells in G_0_/G_1_ and S phases were quantified. (c) Annexin V-FITC/PI staining was analyzed from cells mentioned above.* Lower right quadrant* represents Annexin V^+^/PI^−^ cells (early apoptotic cells).* Upper right quadrant* shows Annexin V^+^/PI^+^ cells (late apoptotic cells). (d) Apoptosis assay of percentages of apoptotic (early and late apoptotic) cells was quantified.

To elucidate the mechanism by which CKIP-1 suppressed glioma cell proliferation, we first investigated the effect of inhibition on cell cycle. Overexpressing- and knockdown-CKIP-1 glioma cells were assayed by flow cytometry to determine the cell cycle profiles (Figure 3(a)). After 72 h of transfection, the proportion of U251-CKIP-1 cells in G_0_/G_1_ phase significantly accumulated (from 26.4% to 43.2%), whereas the percentage of cells in S phase decreased sharply (from 17.1% to 8.5%) compared with that of U251-Ctrl cells (all* p* < 0.01; Figure 3(b),* left*). In contrast, CKIP-1 depletion (U87-RNAi) resulted in an accumulation in S phase in greater numbers (from 8.2% to 17.4%) but reduced that in G_0_/G_1_ phase (from 47.7% to 37.0%) than U87-Ctrl cells (all* p* < 0.01; Figure 3(b),* right*). These results demonstrated that CKIP-1 arrested glioma cells at the G_0_/G_1_ phase which control that cells do not exit from G1 phase and enter into S phase of the cell cycle, while depletion of CKIP-1 reversed the accumulation in the G_0_/G_1_ phase and allowed the glioma cells to enter into S phase, suggesting that CKIP-1 may function through its antiproliferative activity in glioma cells.

To determine whether the cell apoptosis occurring in glioma cells regulated by CKIP-1 is a result of G_0_/G_1_ arrest, we performed flow cytometry using Annexin V-FITC/PI assays (Figure 3(c)). As expected, there was a significantly increasing percentage of early (Annexin V^+^/PI^−^) and late apoptotic (Annexin V^+^/PI^+^) U251 cells (from 0.7% to 10.5%) after 72 h of CKIP-1 transfection (*p* < 0.01), whereas the CKIP-1-RNAi decreased a low percentage of apoptotic U87 cells (from 16.3% to 6.6%,* p* < 0.01; Figure 3(d)). Therefore, the findings demonstrated that CKIP-1 induced apoptosis in glioma cells, suggesting that it may function through its proapoptotic activity.

### 3.4. CKIP-1 Inhibited AKT/GSK3*β*/*β*-Catenin Pathway in Glioma Cells

**Figure 4:**
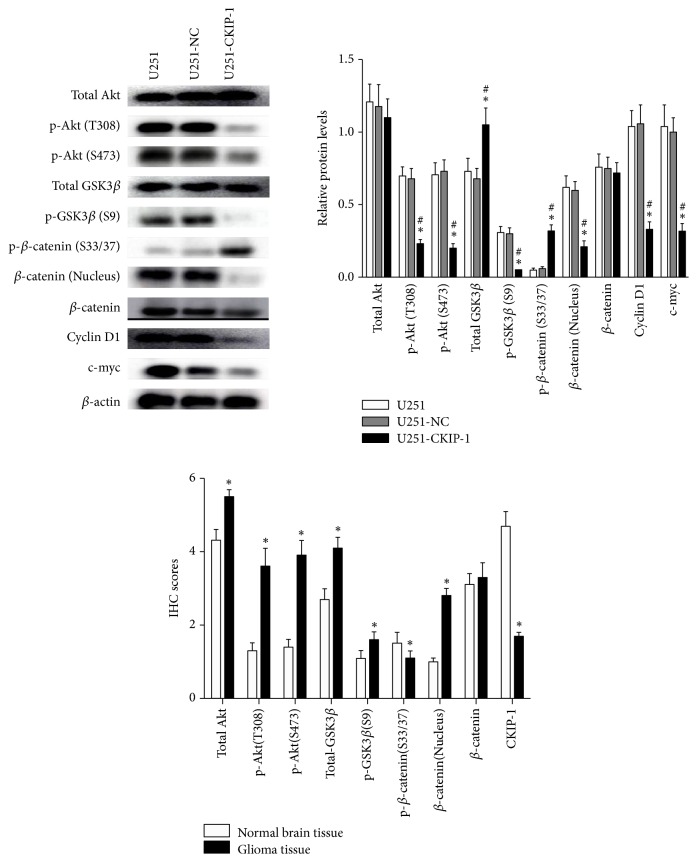
*CKIP-1 inhibited the AKT/GSK3β/β-catenin pathway in U251 cells*. (a-b) Western blot analysis for total Akt, p-Akt (T308), p-Akt (S473), total GSK3*β*, p-GSK3*β* (S9), p-*β*-catenin (S33/37), *β*-catenin (nucleus), *β*-catenin, cyclin D1, and c-myc protein expression in U251, U251-NC, and U251-CKIP-1 cells, respectively. NC: negative control. *∗ p* < 0.05* vs*. U251, ^#^* p* < 0.05* vs*. U251-NC. (c) IHC scores of CKIP-1 and AKT/GSK3*β*/*β*-catenin expression in normal human or glioma patients' brain tissues. △*p* < 0.05* vs*. normal brain tissue.

We next investigated underlying mechanism through which CKIP-1 inhibited glioma tumorigenesis. For this, we analyzed the association between CKIP-1 and candidate signaling pathway proteins by Western analysis (Figures 4(a) and 4(b)). CKIP-1 has been shown to be critical for inhibiting the activation of Akt (protein kinase B) in several types of cancer cells [8]. It interacts with TRAF6 and inhibits TRAF6-mediated Akt activation in macrophage [15]. In addition to Akt, *β*-catenin translocates into the nucleus and binds transcription factor TCF4 and serves as a transcriptional activator, inducing cancer cell proliferation and migration [16]. Phosphorylation of *β*-catenin at S33 and S37 promotes its degradation [17], and its downstream target genes c-myc and cyclin D1 are responsible for tumor proliferation or malignant progression [18]. In this study, we observed phosphorylated-Akt at Thr308 [p-Akt (T308)] and Ser473 [p-Akt (S473)] as well as phosphorylated-GSK3*β* at Ser9 [p-GSK3*β* (S9)] in U251 cells that transfected with CKIP-1 but was expressed at relatively higher levels in U251-Ctrl and parental cells. This result demonstrated that CKIP-1 induced the downregulation of p-Akt (T308), (S473), and p-GSK3*β* (S9). In addition, there was no accumulation of *β*-catenin, but the level of phosphorylated-*β*-catenin at Ser33/37 [p-*β*-catenin (S33/37)] was significantly upregulated. Conversely, the levels of c-myc, cyclin D1, and Smurf1 appeared to decrease by CKIP-1 overexpression. CKIP-1 promotes the self-ubiquitylation and autodegradation of Smurf1. CKIP-1 functions as an inhibitor of Smurf1. We also observed a significant decrease in Smurf1 when CKIP-1 was overexpressed. These results indicated that CKIP-1 may suppress glioma cell proliferation and induce apoptosis through inhibiting the AKT/GSK3*β*/*β*-catenin pathway.

### 3.5. CKIP-1 and AKT/GSK3*β*/*β*-Catenin Expression Scores in Normal Human or Glioma Patients' Brain Tissues

We then used the IHC method to analyze and score CKIP-1 and AKT/GSK3*β*/*β*-catenin proteins in normal brain tissue and patient glioma tissues. From the score we can see that the expression level of CKIP-1 in glioma tissues is lower than that in normal brain tissues, and the activation level of AKT/GSK3*β*/*β*-catenin signaling pathway is significantly higher than that in normal brain tissues. This also indicates that CKIP-1 is inhibited in glioma tissues, while the AKT/GSK3*β*/*β*-catenin signaling pathway is significantly activated (Figure 4(c)).

## 4. Discussion

Gliomas have been considered to be one of the most aggressive and neurologically destructive primary tumors. Therefore, exploring the molecular mechanisms is essential to develop more effective treatment strategies for gliomas. Although the accumulative data have revealed that CKIP-1 was involved in tumorigenesis of multiple cancers and function as a potential tumor suppressor for cancer diagnosis, treatment, and prognosis in the past few years, little is known about its role in the pathophysiology of glioma [19, 20]. Based on glioma tissue microarrays and human U251 glioblastoma cell line, we explored the roles of CKIP-1 in glioma. First, we found that CKIP-1 was significantly downregulated in human gliomas tissues compared with adjacent nontumor brain tissue. The downregulation of CKIP-1 in glioma tissue is associated with worse prognosis in patients with glioma. Second, we showed that upregulation of CKIP-1 suppressed human U251 cell proliferation, colony formation, and invasion and promoted apoptosis in vitro. Third, we revealed that CKIP-1 can negatively regulate Akt activation, which contributes to the tumor-suppressive effect of CKIP-1. In addition, CKIP-1 inhibits the phosphorylation of GSK3*β* and the underlying mechanism may be associated with suppressing the AKT/GSK3b/b-catenin signaling pathway in human U251 glioblastoma cell line.

Glioma is one of the most common primary brain tumors accounting for 30 to 40% of all intracranial tumors. Median survival is less than 12 months for glioblastoma (grade IV) which is the most common and also the most aggressive form of gliomas. Currently, the standard treatment for glioma is surgical resection followed by combination radiotherapy and chemotherapy. It is difficult to completely remove glioma tumor owing to no obvious boundaries from normal brain tissues. This leads to the high recurrence rate of glioma. Therefore, exploring and evaluating new gene targets against glioma cell invasion is a promising approach for treatment of glioma. CKIP-1 was originally identified as a specific interacting protein of casein kinase 2 (CK2) *α* subunit and its role was gradually being unraveled in several human cancers. It has been reported that CKIP-1 expression suppresses osteosarcoma and human epithelial carcinoma formation in nude mice [8]. Historically, there are few clinical researches about the role of CKIP-1 in different tumors. Our study found that the expression of CKIP-1 was lower in high-grade gliomas than low-grade gliomas. In addition, CKIP-1 expression was negatively correlated with glioma clinical grade, TNM stage, and WHO grade but not sex nor age. The survival analysis revealed that low expression levels of CKIP-1 were correlated with a worse prognosis for patients with gliomas. Therefore, CKIP-1 may play important roles in the regulation of invasion and metastasis of glioma cells.

It has been widely demonstrated that CKIP-1 acts as a suppressor in several types of human cancer cells including human osteosarcoma SaOS-2, human epithelial carcinoma A431, gastric cancer cell, colon cancer HCT116, and SW480 cells [8, 20, 21]. However, its actual role and underlying molecular mechanisms in the development of glioma remain unclear. Limitless replicative potential and evasion of apoptosis are the remarkable hallmarks of malignancies. According to our data, overexpression of CKIP-1 significantly inhibited proliferation and induced G1/S arrest and apoptosis of U251/CKIP-1 cells. Invasion and metastasis are the main features of cancer cells which breach the basement membrane barriers and penetrate into the surrounding tissues. Recently, CKIP-1 expression was reported to be related with the invasive capabilities of cancer cells such as human lung cancer cell, gastric cancer cell, and colon cancer cell. Consistent with previous research results, the transwell invasion assay in this study indicated that CKIP-1 overexpression can suppress the invasive ability of human glioma cells.

The Wnt/*β*-catenin pathway is intensively studied in multiple cancers, but its role in glioma has only started to emerge [22–24]. It has been reported that knockdown of CKIP-1 markedly upregulated the phosphorylated Akt levels at Thr308 and Ser473 and the phosphorylation of GSK-3*β* [15]. This indicates that CKIP-1 interacts and regulates the activity of Akt. Another study showed that CKIP-1 interacts with PH domain of each Akt isoform (Akt1, Akt2, and Akt3) via its NH2 terminus by GST pull-down assay [8]. Similarly, in a murine macrophage cell line, RAW264.7 cells, depletion of CKIP-1 by shRNA resulted in sustained activation of Akt-GSK3*β* signaling upon M-CSF treatment [8] while overexpression of CKIP-1 dramatically inhibited Akt activation by membrane recruitment and TRAF6-mediated Akt ubiquitination. Our results showed that overexpression of CKIP-1 did not significantly change the total level of Akt in U251 cell. However, western blot analysis revealed that the phosphorylation levels of Akt (Thr308 and Ser473) were dramatically decreased in U251/pcCKIP-1 compared with U251 and U251/pcNC cells. Moreover, overexpression of CKIP-1 markedly downregulated the phosphorylated GSK3*β* at Ser9 and upregulated the phosphorylated *β*-catenin (Ser33/37) which leads to the ubiquitylation and proteasomal degradation of *β*-catenin [15]. In this study, our results verified the interaction between CKIP-1 and AKT and the effects of CKIP-1 on AKT/GSK3*β*/*β*-catenin signaling pathway in glioma cell. These results suggested an essential role for CKIP-1 in regulation of biological behavior of glioma cell.

In summary, we demonstrated that the CKIP-1 is commonly downregulated in glioma tissues. Lower expressed CKIP-1 promotes glioma cell proliferation and patients with low level of CKIP-1 are associated with a poor prognosis. Our results also showed that upregulation of CKIP-1 inhibits the proliferation of human glioma cell line via suppression of AKT/GSK3*β*/*β*-catenin signaling pathway. These findings suggested that CKIP-1 might be a potential therapeutic target in the treatment of glioma.

## Figures and Tables

**Table 1:** Correlation between the CKIP-1 protein expression and clinicopathological factors of glioma patients.

Clinicopathological factors	No. of patients	CKIP-1 protein expression		*p* value
		Low	High	
Age (year)				
< 40	28	22	6	0.812
≥ 40	59	45	14	
Sex				
Male	45	34	11	0.738
Female	42	33	9	
Tumor location				
Frontal	36	27	9	0.834
Parietal	12	10	2	
Occipital	10	8	2	
Temporal	29	22	7	
Tumor diameter				
< 4 cm	40	29	11	0.356
> 4 cm	47	38	9	
WHO grade				
I	3	0	3	0.001
II	16	4	12	
III	27	21	6	
IV	41	39	2	

## Data Availability

The data used to support the findings of this study are available from the corresponding author upon request.
